# Cigarette and waterpipe smoking among adult patients with severe and persistent mental illness in Bahrain: a comparison with the National Non-communicable Diseases Risk Factors Survey

**DOI:** 10.1186/s13104-016-1894-9

**Published:** 2016-02-09

**Authors:** Randah R. Hamadeh, Ahmed Al Ansari, Haitham Jahrami, Adel Al Offi

**Affiliations:** College of Medicine and Medical Sciences, Arabian Gulf University, P.O. Box 26671, Manama, Kingdom of Bahrain; Psychiatric Hospital, Ministry of Health, P.O. Box 5128, Manama, Kingdom of Bahrain

**Keywords:** Mental illness, Smoking, Waterpipe, Prevalence, Bahrain

## Abstract

**Background:**

Smoking has been associated with several types of mental illness namely schizophrenia, depression, bipolar disorders with a prevalence of smoking twice that of the general population. The study objective was to ascertain whether waterpipe tobacco smoking (WTS), cigarette smoking and all types of tobacco smoking are more common among Bahraini patients with severe and persistent mental illness (SPMI) than the general population.

**Methods:**

A cross-sectional study was conducted on 222 adult SPMI both in- and out- patients who attended the Psychiatric Hospital in Bahrain. A 29-item questionnaire, which included sociodemographic variables, pattern and history of psychiatric illness and a comprehensive smoking history, was used. Comparative smoking data were obtained from the Bahraini National Non-communicable Diseases Risk Factors Survey.

**Results:**

The prevalence of smoking of tobacco among SPMI patients was 30.2 % compared to 19.9 % in the general population. The corresponding values for cigarette smoking were 25.2, 13.8 %, respectively and for WTS, 11.3, 8.4 %, respectively. SPMI patients were 1.7 (95 % CI 1.3, 2.4 %) times more likely to be smokers, 2.1 (95 % CI 1.5, 2.9 %) times, cigarette smokers and 1.4 (95 % CI 0.9, 1.9 %) times WTS than the general population. SPMI patients smoked at a younger age and consumed more cigarettes than the general population. The mean age started smoking was lower among men than women, similar for cigarettes, and higher for WTS.

**Conclusions:**

The prevalence of smoking among patients with SPMI in Bahrain is twice that of the general population. The findings of the study have implications on the provision of healthcare to mentally ill patients in the country.

## Background

Smoking is a major determinant of global health. In 2013, it occupied the second rank among the global risk factors, accounted for 6.1 million deaths and 143.5 million DALYs [1, 2]. It is a major risk factor for several non-communicable and respiratory diseases and substantially affects the health of fetuses of pregnant women and children exposed to tobacco smoke [3]. Most of the evidence of the effects of smoking is based on cigarette smoking, however the health effects of waterpipe tobacco smoking (WTS) became evident in recent years [4, 5].

Although the smoking prevalence has decreased over the past few decades in the general population in most countries, it remains a public health priority as the number of smokers increased significantly as a result of population growth and disproportionately high for distinct groups such as those with severe mental illness [1, 6–8].

Smoking has been associated with several types of mental illness namely schizophrenia, depression, anxiety disorders, bipolar disorders and dementia. The prevalence of smoking among those with mental illness is twice that of the general population [7, 9]. Moreover, patients with coronary heart disease were reported to have high levels of depression highlighting the importance of tobacco control in mental illness [10]. Primack and colleagues reported that the associations of mental health problems were stronger for cigarette smoking than WTS [11]. In the United States (US), nearly 1 in 5 adults have some form of mental illness, of whom 36 % smoke cigarettes. In comparison, 21 % of US adults without mental illness smoke cigarettes [12]. Notably, the rate of tobacco use increases three times in the psychiatric population, and in particular, a range of 40–100 % is found among schizophrenics. Up to 90 % of those with schizophrenia smoke cigarettes [13, 14]. Accordingly, smokers with schizophrenia are 30 % more likely than smokers in the general population to die from cardiovascular disease and 60 % more likely to die from respiratory disease [15, 16]. The average life expectancy for individuals with schizophrenia is 20 % lower than the general population [17]. Schizophrenic patients may perceive the health risks of cigarette smoking to be less than the normal population [18]. Adults with mental illness smoke 70 % of consumed cigarettes in the US [19]. 40 % of men and 34 % of women with mental illness smoke. Additionally, 48 % of people with mental illness who live below the poverty level smoke, compared with 33 % of those with mental illness who live above the poverty level [12].

Tobacco smoking has attracted the attention of mental health professionals worldwide in recent years. Experts attribute the link between smoking and mental illness to a number of factors, which include the biochemistry of tobacco and nicotine, the nature of mental illnesses and the culture of mental health system [9]. However, most of the research that explored the association between tobacco smoking and mental illness was based on cigarette smoking and not WTS [20].

Little published work has focused on determining the prevalence of tobacco smoking among patients with mental illnesses in Arab and other Middle Eastern countries [21, 22] and its comparison to that of the general population. An Egyptian study found no difference in smoking rate among schizophrenic patients with or without obsessive–compulsive symptoms [22]. A recent study from a center in north Iraq reported higher smoking rates for schizophrenic patients compared to other mental disorders and that they had smoked earlier than appearance of their first symptoms [23]. A research from Saudi Arabia reported a prevalence rate of 57.9 % among the male outpatient population compared to 78 % of psychiatric inpatient in Iran [21, 24]. The prevalence rates of smoking in Saudi patients with different diagnoses were as follows: schizophrenia 56.5 %, major depressive disorder (MDD) 59.6 %, anxiety disorders 53.0 % and substance abuse 72.0 % [21]. These studies focused on specific psychiatric populations, from limited geographic areas and did not include community population or national smoking statistics.

The Bahraini National Non-communicable Diseases Risk Factors Survey (NNDRFS) in 2007 reported that the prevalence of smoking of all types of tobacco in males, females and both sexes combined in Bahraini nationals was 33.4, 7.0 and 19.9 %, respectively [25]. The corresponding figures for cigarette smoking were 27.0, 1.2 and 13.8 %, respectively and that of WTS, 10.8, 6.2 and 8.4 %, respectively. No previous studies have been under taken in Bahrain to examine the prevalence of smoking among patients with mental illness.

The Psychiatric Hospital in Bahrain is the main and only public services facility for mental illness in the country. In 2012, 405 patients were admitted to the Psychiatric Hospital in the age group 15–64 years. 232 were diagnosed with schizophrenia, 97 bipolar affective disorder (BAD) and 77 MDD according to the Ministry of Health’s 2012 Annual Report [26]. The objective of this study was to ascertain whether WTS, cigarette smoking and all types of tobacco smoking are more common among Bahraini patients with severe and persistent mental illness (SPMI) than the general population (NNDRFS).

## Methods

A cross sectional study was conducted on adult (20–64 years) Bahraini outpatients and inpatients who attended the Psychiatric Hospital in Bahrain with SPMI from 1 November 2014 to 31 January 2015.

The Psychiatric Hospital was founded in 1932. The hospital has 246 inpatient beds (16-beds for alcohol and drug dependence patients, 55 for acute patients, 40 for short-stay patients, 10 for child psychiatry, 80 for long-stay patients and 45 for the intellectual disabilities). The Psychiatric Hospital is considered to be the main center for psychiatric care services in the country. The hospital works for 24 h and accepts psychiatric disorder and social cases which come directly or are referred from other hospitals and health centers.

A convenient sample of 240 SPMI patients, which included schizophrenia, BAD and MDD classified according to the International Classification of Disease (ICD 10) criteria was selected. SPMI patients whose mental status did not allow them to be interviewed or had comorbidity with other psychiatric diagnoses were excluded from the study. Convenience non-probabilistic sampling technique was the most applicable and feasible technique in this research due to the nature of the research topic. The sample size was determined using the prevalence of smoking in Bahrain (19.9 %) based on that reported in the NNDRFS, alpha of 0.05 and a beta of 0.80. This resulted in a minimum sample size of 210 patients. Data collection included all of the general adult psychiatric clinics in the hospital on all working days of the week during the 2 months data collection period. Eighteen patients refused to participate in the study mostly due to transport related matters and 222 accepted.

Two independent, trained hospital staff collected the data for this research during a semi-structured interview session. These patients were regularly following up their treatment appointments with psychiatrists, and had been seen on many previous occasions by a multidisciplinary team, thus comprehensive data had been recorded in their medical record files. These files were reviewed for abstracting socio-demographic characteristics, diagnosis, and clinical history. In addition, the data collection staff interviewed the patients privately and recorded their responses using a 29-item questionnaire, which included questions on smoking history taken from the NNDRFS. The latter used face-to-face interviews similar to the data collection method of the current study. Smokers were defined according to those used in the NNDRFS to allow comparisons. Smoking status was categorized into current daily, current occasional, ex- and non-smokers. Smokers were classified according to tobacco types as cigarette (manufactured and hand-rolled), *shisha* (waterpipe) or other types (cigar or pipe) smokers. Average number of cigarettes smoked daily was calculated for daily smokers only [25].

The researchers briefed each patient as well as key relatives regarding this study. Participating patients gave their consent prior to interview and those who refused were excluded.

Data was entered and analyzed using the SPSS version 22.0. Chi-square test, independent samples *t* test and ANOVA were used where applicable. The p value was considered significant at <0.05. The standardized smoking prevalence ratios (SSPR) were calculated similar to the method used to calculate standardized mortality ratios. To get the expected number of smokers, the age specific smoking rates from each age group in the standard population were multiplied by the corresponding number of SPMI in that age group. The total expected number of smokers was obtained by summing the expected numbers of smokers in each age group. The observed total number of smokers among the SPMI was divided by the expected number of smokers and then multiplied by 100 to get SSPR. This was repeated for all types of smoking, cigarettes and WTS. The corresponding 95 % confidence intervals were calculated for SSPR.

Ethical approval was attained from the Research Technical and Support Committee, Ministry of Health, Kingdom of Bahrain.

## Results

Of the 222 SPMI patients (46 inpatients and 176 outpatients), there were 99 with schizophrenia (44.6 %), 86 BAD (38.7 %) and 37 (16.7 %) MDD. Table 1 show that the majority of the patients were in the age groups 40–49 and 30–39. The SPMI patients were almost similar with respect to gender but there was a significantly lower percentage (p = 0.002) of females among schizophrenia, half that of MDD. About 40.0 % of SPMI patients had less than a high school degree and over half had a monthly family income of 150–500 BD (398–1327 US $). Only 21.2 % of SPMI patients were employed with statistically significant (p < 0.0001) differences between the patients of the three clinical categories.

**Table 1:** Sociodemographic characteristics of SPMI patients

	Schizophrenia (n = 99)		MDD (n = 86)		BAD (n = 37)		Total (n = 222)	
	No.	%	No.	%	No.	%	No.	%
Gender*								
Male	61	61.6	31	36.0	17	45.9	109	49.1
Female	38	38.4	55	64.0	20	54.1	113	50.9
Age								
20–29	18	18.2	13	15.1	9	24.3	40	18.0
30–39	27	27.2	14	16.3	9	24.3	50	22.5
40–49	25	25.3	27	31.4	9	24.3	61	27.5
50–59	17	17.2	16	18.6	7	18.9	40	18.0
60–64	12	12.1	16	18.6	3	8.2	31	14.0
Education								
Illiterate	9	9.1	6	7.0	2	5.4	17	7.7
Read and write	7	7.1	6	7.0	1	2.7	14	6.2
Primary	12	12.1	7	8.0	4	10.8	23	10.4
Intermediate	17	17.2	12	14.0	6	16.2	35	15.8
High school	40	40.4	35	40.7	15	40.5	90	40.5
University	14	14.1	20	23.3	9	24.4	43	19.4
Income (BD)								
<150	28	28.3	13	15.1	7	18.9	48	21.6
150–500	45	45.5	50	58.1	19	51.4	114	51.4
>500	26	26.3	23	26.7	11	29.7	60	27.0
Employment**								
Employed	14	14.2	22	25.6	11	29.7	47	21.2
Not employed	53	53.5	17	19.8	9	24.3	79	35.6
Housewife	18	18.1	32	37.2	8	21.6	58	26.1
Retired	14	14.2	15	17.4	9	24.3	38	17.1

* p < 0.005; ** p < 0.0001

Sixty eight percent of SPMI patients were diagnosed for over 5 years. The corresponding proportions for schizophrenia, BAD and MDD were 79.8, 55.8, 64.0 %, respectively (p < 0.0001). The mean outpatient visits during 2014 of SPMI patients were 6.9 (95 % CI 6.3, 7.5) with slight variation between the diagnoses (schizophrenia, 7.3 (95 % CI 6.2, 8.3), MDD 6.6 (95 % CI 5.7, 7.5) and BAD 6.6 (95 % CI 5.1, 8.2). There was a statistically significant difference in the number of admissions to the Psychiatric Hospital (p < 0.0001) with over one third of schizophrenic patients (35.4 %) having over six admissions in the past 5 years compared to 5.8 % of BAD, 27.0 % MDD and 22.5 % for all SPMI patients.

Almost one third of the SPMI patients were smokers (30.2 %, 95 % CI 24.2, 36.7 %). The prevalence of smoking was higher in men (47.7 %, 95 % CI 38.1, 57.5 %) compared to 13.3 % for women (Table 2). Cigarette smoking (46.8 %, 95 % CI 37.2, 56.6 %) was more popular than WTS (11.0 %, 95 % CI 5.8.1, 18.4 %) among men. In contrast, WTS (11.5 %, 95 % CI 6.3, 18.9 %) was almost double that of cigarette smoking (4.4 %, 95 % CI 1.4, 10.0 %) among women. The prevalence of WTS was similar in both sexes. The differences among the clinical categories with respect to smoking were not significant except for cigarette smoking in both genders. In both sexes combined and among men, BAD had the highest prevalence of smoking any type of tobacco (35.1 %, 95 % CI 20.2, 52.5 and 58.8 %, 95 % CI 32.9, 81.6 %, respectively).

**Table 2:** Prevalence of smoking of SPMI patients by diagnosis, gender and type

Type	Schizophrenia	MDD	BAD	Total^a^	p value
Male					
Cigarettes	49.2 (36.1, 62.3)	35.5 (19.2, 54.6)	58.8 (32.9, 81.6)	46.8 (37.2, 56.6)	0.445
Waterpipe	9.8 (3.7, 20.2)	16.1 (5.5, 33.7)	5.9 (0.1, 28.7)	11.0 (5.8, 18.4)	0.504
Any type	49.2 (36.1, 62.3)	38.7 (21.9, 57.8)	58.8 (32.9, 81.6)	47.7 (38.1, 57.5)	0.387
Female					
Cigarettes	2.6 (0.1, 13.8)	5.5 (1.1, 1.5)	5.0 (1.3, 24.9)	4.4 (1.4, 10.0)	0.802
Waterpipe	5.3 (0.6, 17.7)	14.5 (6.5, 26.7)	15.0 (3.2, 37.9)	11.5 (6.3, 18.9)	0.334
Any type	7.9 (1.7, 21.4)	16.4 (7.8, 28.8)	15.0 (3.2, 37.9)	13.3 (7.6, 20.9)	0.481
Both sexes					
Cigarettes	31.3 (22.4, 41.4)	16.3 (9.2, 25.8)	29.7 (15.9, 47.0)	25.2 (19.7, 31.5)	0.050
Waterpipe	8.1 (3.6, 15.3)	15.1 (8.3, 24.5)	10.8 (3.0, 25.4)	11.3 (7.4, 16.2)	0.318
Any type	33.3 (24.2, 43.5)	24.4 (15.8, 34.9)	35.1 (20.2, 52.5)	30.2 (24.2, 36.7)	0.324

^a^95 % CI

SPMI had higher proportions of ever tobacco smokers in males (83.5 %), females (3.2 %) and both sexes combined (57.2 %) than those reported in the NNDRFS (48.7, 1.1, 29.5 %, respectively). The percentages for former smokers of any type of tobacco in SPMI males (35.8 %), females (18.5 %) and both sexes combined (27.0 %) were also higher than in the NNDRFS (15.3, 4.1, 9.6 %, respectively). The prevalence of current smoking of any type of tobacco among SPMI patients was 30.2 % compared to 19.9 % in the general population (p = 0.001). The corresponding values for cigarette smoking were 25.2 %, 13.8, respectively (p < 0.0001) and for WTS 11.3, 8.4 %, respectively. 62.7 % of smokers among SPMI patients smoked cigarettes only compared to 53.7 % of the population, 16.4 % WTS only compared to 26.4, 20.9 % mixed compared to 15.9 %. None of the SPMI smokers smoked cigar or pipe compared to 4.0 % of the general population.

Male SPMI patients were 1.8 times more likely to be smokers and 2.4 times to be cigarette smokers than the general population but similar with respect to WTS. As for women patients with SPMI, they were 2.0 times more likely to be smokers and WTS and 3.8 times to be cigarette smokers than the general population. The odds ratios for both sexes combined were 1.7, 2.1 and 1.4, respectively (Table 3). The SSPR for cigarette smoking among SPMI patients was 190 (95 % CI 144, 247 %). The corresponding SSPRs for WTS and all type of smoking were 85 (95 % CI 55, 126 %) and 153 (95 % CI 119, 194 %), respectively. All SPMI patients who smoked cigarettes were daily smokers compared to 96.3 % of the cigarette smokers in the population. As for WTS, 45.6 % were occasional smokers in the population compared to none among SPMI patients. Further, a higher percentage of SPMI patients were former smokers (27.0 %) than the population (9.6 %). The corresponding proportions among men were 35.8, 15.3 %, respectively and women 18.5, 4.1 %, respectively.

**Table 3:** Prevalence rates of smoking by gender and type: comparison with the general population

	SPMI patients	General population	OR (95 %CI)
Male			
Cigarettes	46.8	27.1	2.4 (1.6, 3.5)
Waterpipe	11.0	10.8	1.0 (0.5, 1.9)
Any type	47.7	33.4	1.8 (1.2, 2.7)
Female			
Cigarettes	4.4	1.2	3.8 (1.3, 11.0)
Waterpipe	11.5	6.2	2.0 (1.0, 3.7)
Any type	13.3	7.1	2.0 (1.1, 3.7)
Both sexes			
Cigarettes	25.2	13.8	2.1 (1.5, 2.9)
Waterpipe	11.2	8.4	1.4 (0.9, 1.9)
Any type	30.2	19.9	1.7 (1.3, 2.4)

The mean age started smoking any tobacco was lower among men (15.9 ± 0.5 years) than women (18.0 ± 7.3 years), similar for cigarettes (15.9 ± 0.5 and 16.0 ± 6.4, respectively) but higher for WTS (24.2 ± 8.9 and 18.8 ± 7.7, respectively (Table 4). The differences between the SPMI categories were not statistically significant. Further, the mean age at starting to smoke among SPMI patients was lower than the general population for all age groups but the differences were statistically significant only for the age group 20–29 years. MDD patients started to smoke at a younger age (15.3 ± 5.1) than schizophrenics (16.2 ± 3.8) and BAD (18.5 ± 5.2). Moreover, SPMI patients started cigarette smoking at a younger age than the general population in both sexes (Table 5).

**Table 4:** Mean age started smoking by tobacco type and mental disorder

Mental disorder	Cigarette smoking (n = 56)				WP smoking (n = 25)				All types (n = 67)			
	Mean	SD	95 %	CI	Mean	SD	95 %	CI	Mean	SD	95 %	CI
Schizophrenia	16.0	3.8	14.9	17.4	22.3	8.1	15.5	29.0	16.2	3.8	14.9	17.6
MDD	15.0	3.9	12.8	17.2	18.8	9.2	13.3	24.4	15.3	5.1	13.0	17.7
BAD	16.9	3.5	14.5	19.3	27.8	3.2	22.7	32.8	18.5	5.2	15.4	21.7
Total	15.9	3.7	14.9	16.9	21.4	8.5	17.8	24.9	16.4	4.6	15.3	17.5
F	0.804				1.844				2.062			
p value	0.453				0.182				0.136

**Table 5:** Mean age started cigarette smoking of SPMI patients by gender: comparison with the general population

	Number	Mean	Std. deviation	p value
Male				
SPMI	51	30.5	19.7	<0.0001
NNDRFS	224	19.4	12.5	
Female				
SPMI	5	23.0	16.4	0.170
NNDRFS	11	10.2	7.2	
Both sexes				
SPMI	56	29.8	19.5	<0.0001
NNDRFS	235	18.9	12.5	

Male, female and both sexes combined of SPMI patients consumed higher number of cigarettes daily compared to the general population (Table 6). There were no statistically significant differences among the SPMI categories with respect to tobacco consumption. The average number of cigarettes per day was highest among schizophrenia patients in men, women and both sexes and MDD for WTS per week.

**Table 6:** Average number of cigarettes smoked daily by SPMI patients and the general population by gender

Gender	SPMI	General population	p value
Male	30.5.4 ± 19.7	19.4 ± 12.5	<0.0001
Female	23.0 ± 16.4	10.2 ± 7.2	0.170
Both sexes	29.8 ± 19.5	18.9 ± 12.5	<0.0001

## Discussion

One third of SPMI in both sexes combined and half of the males were smokers. The number of smokers among SPMI was twice that of the general population for all types. This finding is consistent with some reported studies [7, 27, 28]. Such high prevalence of smoking among SPMI patients would increase the risk of smoking related health complications and death from vascular diseases, cancer and other disease conditions [29, 30]. Article 4 of the Bahrain 2009 Antismoking Law prohibits tobacco smoking in closed public places including health establishments; however the majority of the SPMI in the study was outpatients and could smoke freely outside the hospital premises. As for inpatients they generally smoked outside the building but for those who were unable to go out (none of the study population were in this category) they have an allocated room for them to smoke. This raises the question whether the Psychiatric Hospital should continue to permit smoking for those patients irrespective of the law or completely prohibit it [31, 32].

WTS among SPMI patients was 1.5 times that of the general population. Comparative data of WTS among mentally ill patients in the region is scarce as WTS and mental illness was not well researched in the region. It is noteworthy that Nejad and Pouga reported similar figures of hookah smoking rate among inpatients with SPMI in Iran [33]. The finding that cigarette smoking was higher in both sexes among SPMI compared to the population is expected. Given that SPMI use tobacco more than the general population, it is not surprising that SPMI women had higher rates of WTS as it is more culturally acceptable for women to smoke waterpipe than cigarettes. In contrast, any type of tobacco use by men is socially tolerated. Further, the higher daily consumption of cigarettes by SPMI patients compared to the general population is consistent with the literature as these patients tend to use cigarettes to alleviate their symptoms [33].

Another important finding was that more than one third of male SPMI patients who ever smoked (35.8 %) quit smoking compared to 15.3 % from the same gender in the general population. This indicates even a higher smoking prevalence among this vulnerable population. Cigarette smoking was higher among schizophrenia and BAD patients compared to those with MDD (p = 0.050). SPMI patients may use smoking as a way of self-medication to alleviate symptoms and improve cognition [33, 34].

Furthermore, high smoking rates among SPMI patients support some theories that try to explain why mentally ill patients smoke more than the general population. It has been reported that nicotine derivatives interfere with the metabolism of some of the antipsychotic medication and can affect blood drug levels [35, 36]. It could be that psychotic patients smoke in order to reduce the effect of these medications and their side effects. The finding that SPMI patients smoke at a younger age and consume more cigarettes than the general population further supports studies from other parts of the world [7].

The study had some limitations as the sample included patients with long mental illness history and frequent hospital admissions, which might make them possibly different from SPMI patients in the community. Further, the prevalence of smoking in the general population was based on those of 2007; the only available data and the time lag between NNDRFS and this study cannot be overlooked. Finally, the generalizability of the findings is limited since convenience sampling in the selection of the study population was used.

The major strength of this study is that it is the first of its kind in the Eastern Mediterranean Region as it compares mentally ill patients with the general population with respect to cigarette smoking, WTS and all types of tobacco smoking in both sexes.

The implication of the research results on policy and practice at the Psychiatric Hospital include abolishing the use of cigarettes as an incentive in long stay wards, introducing smoking related questions in the personal history of patients, adding the subject of smoking to the psycho-education program of schizophrenic patients and providing smoking cessation assistance to smokers. Smokers can be referred by their psychiatrists to the Quit Tobacco Clinics (QTC) as these clinics are available free of charge to any smoker intending to quit or has been referred by the physician. Further, the Ministry of Health might consider opening a QTC at the Psychiatric hospital as the QTC are located in the primary healthcare centers of the ministry.

## Conclusions

The findings of this study highlight the importance of smoking prevention and cessation programmes. All efforts should be mobilized to strengthen tobacco control measures in the country and modify the hospital policies and regulations to control tobacco use. Moreover, including tobacco use in personal history and providing smoking cessation assistance to smokers needs to be seriously considered. Further, the practice of giving tobacco as an incentive should be stopped. In addition, fostering healthy lifestyle for patients with mental illness should be part of their management.

## Abbreviations

SPMI: severe and persistent mental Illness
WTS: waterpipe tobacco smoking
NNDRFS: National Non-communicable Diseases Risk Factors Survey
MDD: major depressive disorder
BAD: bipolar affective disorder
ICD 10: international classification of diseases version 10
SSPR: standardized smoking prevalence ratios
